# Investigation of the association between quality of life and depressive symptoms during postpartum period: a correlational study

**DOI:** 10.1186/s12905-017-0473-0

**Published:** 2017-11-21

**Authors:** Maria Papamarkou, Pavlos Sarafis, Charis P. Kaite, Maria Malliarou, Andreas Tsounis, Dimitris Niakas

**Affiliations:** 1 Hellenic Open University, 18 Aristotelous str., 26335 Patra, Patras, Greece; 2 Department of Nursing, School of Health Sciences, 15 Vragadinou str, 3041 Limassol, Cyprus; 3 Aristotle University of Thessaloniki, School of Psychology, Thessaloniki, Greece

**Keywords:** Post-partum depression, Quality of life, Women, Sf-36

## Abstract

**Background:**

The onset of a major depressive episode is experienced by a large number of women in the weeks or months following delivery. Postpartum depression may deem those women experiencing it incapable of taking care for themselves, their family and their infants, while at the same time it could negatively affect their quality of life. The present study assessed the quality of life of a sample of mothers in Greece, in order to investigate the association between postpartum depression and quality of life (QoL).

**Methods:**

145 women in a Private-General Obstetrics and Pediatric Clinic in Greece completed the Edinburgh Postnatal Depression scale (EPDS) and SF-36 questionnaire on the third and fourth day after delivery (caesarean or normal childbirth). The data were analyzed using SPSS version 17.0. Linear and logistic regression analysis was performed in order to find the independent factors related to the quality of life and postpartum depression symptoms.

**Results:**

9.9% of the participants experienced postpartum depression symptoms. Significant associations were found between the place of residence and symptoms of postpartum depression, and more specifically, women outside of Attica indicated higher levels of postpartum depression symptoms (*p* = 0.008) than women living in Attica. The level of education was also found to be significantly associated with postpartum depression symptoms, since women with Primary and Secondary education experienced higher levels of postpartum depression symptoms (*p* = 0.005) than those with a tertiary education. Concerning quality of life, women with postpartum depression symptoms scored 24.27 lower in «Role-Physical», 15.60 lower in «Bodily pain», 11.45 lower in «General Health», 14.18 lower in dimension of «Vitality», 38.25 lower in Role – Emotional and 16.82 lower in dimension of mental health, compared to those without depression symptoms.

**Conclusion:**

Postpartum depression symptoms are associated with the quality of life of women after pregnancy, and therefore constitute a powerful predictor of the quality of life. Health care professionals should provide individualized care for the prevention and treatment of Postpartum Depression symptoms in order to help women improve their quality of life.

## Background

Postpartum depression (PPD) is considered to be a common psychiatric disorder after childbirth [1]. Potential risk factors include: a) a previous psychiatric history, b) depression during pregnancy, c) socio-economic insufficiency and d) existence of other medical conditions [2–4]. Postpartum depressive symptoms include amongst others: a) depressed mood, b) weakness, c) disappointment, d) agitation, e) psychological distress, f) sleep disorders [5]. PPD’s prevalence is considered to be high, although rates among different countries vary [6]. In a literature review concerning cross-cultural and social diversity of PPD’s prevalence, in which rates from 143 studies in 40 countries were reported, show that results from different studies raging from almost 0% to almost 60% [6]. In this context, the European Union Committee on Public Health has declared that women with PPD are one of the most important target groups for preventive interventions in the field of depression and promotion of resilience in general health policies [7]. PPD’s consequences are detrimental for both the baby and the mother. On the one hand it can cause delays in children physical, social and cognitive development [8], and on the other, aspects concerning maternity care, and mother’s health and quality of life may be strongly affected [9].

Quality of life (QoL), is according to the World Health Organization definition ([10], p.1570) “the individual’s perception of their life in the context of the culture and value systems in which they live and in relation to their goals, expectations, standards, and concerns”. Previous studies [11, 12], revealed significant correlations between postpartum depression and quality of life. In addition, in a study in Canada, Da costa et al. [13] showed that women with PPD scored significantly lower on all domains of SF-36 in comparison to women without PPD, although there was no association between the severities of depressed mood to worse physical health status. However, the influence of postpartum depression on quality of life or the health status of mothers after birth still remains vague [14]. Several other studies [15, 16], investigated the association of specific types of delivery and QoL.

However, QoL is a concept affected by culture and social systems [12, 17]. It is a term that is used in many studies, mainly in the health care field [18]. Thus it is important to assess the quality of life of a sample of mothers in Greece, in order to investigate the association between postpartum depression and quality of life (QoL) in the aforementioned context. There is a plethora of studies conducted in other cultures. Postpartum period is accompanied by many physical, emotional, and social changes in women’s health. Health professionals should pay close attention in order to avoid unnecessary medical expenses by increased length of stay in clinics. To the authors’ knowledge, no previous study in Greece has measured maternal quality of life during the postpartum period, although PPD’s prevalence and consequences is associated with the cultural differences found in many countries [19]. The present study assessed the quality of life of a sample of mothers in Greece, in order to investigate the association between postpartum depression and quality of life (QoL).

## Methods

### Sample recruitment

A cross-sectional descriptive study among women who gave birth at a private clinic in Athens was conducted. This clinic is a center of excellence in obstetrics and it was purposefully chosen as it was convenient and easily accessible. The data were collected at a period of three months in 2016. During this period, all women who had given birth at the clinic and met all the other inclusion criteria, were approached by members of the research team and were invited to participate in the study. The participants completed the questionnaire at the time of recruitment. Data were collected 3 or 4 days after giving birth because, women are discharged on the fourth day after their admission in the clinic. 205 mothers were invited to participate but only 145 agreed to take part in the study (RR: 70.73%). With the current sample size the study had >90% power to detect significant results through regression analysis. Inclusion criteria were as follows: (a) participants were to be interviewed in their third and fourth day after delivery (caesarean or normal childbirth); (b) participants had to demonstrate the cognitive ability to understand and complete the questionnaires; (c) the mother must have delivered a living, healthy baby and (d) participants had to give their informed consent.

### Informed consent

Belmont Report [20] ethical principles were applied throughout the study. Prior to their participation, all participants signed an informed consent form after being provided with all the details and explanations, in simple language. The study was approved by the Scientific Council of the Private Clinic “Mitera A.E.” in Athens on November 2015 and questionnaires were distributed for a period of 3 months’ in 2016.

The participants were informed that they could voluntarily terminate their participation in the study at any time without any consequences to themselves or to the quality of their health care. All information obtained from research participants was kept confidential.

### Instruments

An anonymous self-administered questionnaire was used. The first part contained questions recording socio-demographic, social and health-related characteristics of the sample including age, marital status, place of living, educational level, occupational status, one statement concerning their relationship with their husband and the existence or not of any chronic disease.

Participants completed the Greek version of the Edinburgh Postnatal Depression Scale (EPDS) [21]. EPDS has been chosen as it has been developed in screening postpartum depression [22] and has been widely used for that purpose in numerous other studies [4, 12, 13, 23, 24]. EPDS consists of 10 points with a four-item scale, with maximum scores of 30. The Greek version of the EPDS was found to be a valid and reliable instrument with an internal consistency of a = 0.9. Statistical analysis suggested that a cut-off point of 11/12 with sensitivity 90% and specificity 97.2% [21]. Therefore, a total score of 12 or greater has been recommend as an indicator of possible depression [21].

On the other hand, in the current study, the Greek version of Medical outcomes study-short form (SF-36), was also used [25, 26]. SF-36 is considered to be a valid and reliable tool with high convergent and discriminatory validity that distinguishes between groups of respondents based on their age, gender and socioeconomic status [26]. It consists of 36 items with eight sub-scales: Physical Functioning (PF), role limitation due to physical problems or role-physical (RP), bodily pain (BP), general health (GH), vitality (VT), social functioning (SF), role limitation due to emotional problems or role-emotional (RE), and emotional wellbeing (EW). Each subscale ranges from zero to 100 with higher scores indicating a better condition. SF-36 is also divided into two components: Physical health (based on the PF, RP, BP and GH scales) and mental health (relating to VT, SF, RE and EW) [4]. The SF-36 was selected as a measure for our study since according to the literature [9] is the most frequently used tool in the relevant studies assessing the relationship between PPD and quality of life. In addition, SF-36 is a generic instrument, whose relationship with quality of life is more debatable, while there are few quality of life tools that has been specifically designed for the maternity care setting [9], from which, to our knowledge, none have been tested for their validity and reliability properties to the Greek population.

### Analysis

Mean values and Standard Deviation were used for the description of the quantitative variables. Absolute (N) and the relative (%) frequencies were used for the description of quantitative variables. For comparison of proportions Pearson’s and Fisher’s exact tests were used. For comparison of quantitative variables between two groups Student’s t-test was used. Linear regression analysis with a stepwise method (p for entry 0.05, p for removal 0.10) was used in order to find independent factors associated with SF-36 dimensions. Kolmogorov-Smirnov test was used to investigate if the normality assumption was satisfied. General health was log-transformed due to its skewed distribution. The significance levels are two-sided and statistical significance was set at 0.05. Data were analyzed using SPSS version 17.0.

## Results

The mean age of the participants was 33.3 years (±4.2). Sample characteristics are presented in Table 1. The majority of them (84.5%), where living in Attica and were Greek (96.5%), 97.2% were married, most of them (47.9%) were employees working in the private sector and two women had a chronic disease. Additionally, 17% of the participants declared not having a very good relationship with their husband. (Table 1).

**Table 1:** Sociodemographics, Social and Health-related Characteristics of the Participants

		Mean ± SD/Age range	Number	Percent
Age(years)				
		33.3 ± 4.2/23–45		
Marital status	married		141	97.2
	single		3	2.1
	divorced		1	0.7
Place of living	Not in Attica		22	15.5
	In Attica		120	84.5
Nationality	Greek		139	96.5
	Other		5	3.5
Education	High school		33	22.8
	Higher education (university)		91	62.8
	Master’s		21	14.5
Occupation	Household		9	6.3
	Unemployed		14	9.7
	Public Servant		23	16.0
	Private Employee		69	47.9
	Merchants, tradesmen		16	11.1
	other		13	9.0
Relationship with husband	Bad		5	3.5
	Moderate		2	1.4
	Good		17	12.1
	Very good		117	83.0
Having a chronic disease	Yes		2	1.4
	No		140	96.6

9.9% (*N* = 14) of the women were found to score high on EPDS (greater than cut-off 12). Association of postpartum depression with demographics and other characteristics is shown in Table 2. Significant correlations were found between the place of residence and symptoms of postpartum depression and more specific women outside of Attica indicated higher levels of postpartum depression symptoms (*p* = 0.008) than women living in Attica. The level of education was also found to be significantly correlated with postpartum depression symptoms, since women who did not undertake tertiary education experienced higher levels of postpartum depression symptoms (*p* = 0.005). (Table 2).

**Table 2:** Correlation of postpartum depression symptoms with demographics and other characteristics of the participants

		postpartum depression symptoms				P
		NO		YES		
		N	%	N	%	
Age (years)		33.4 ± 4.2		32.32 ± 4.21		0.343^b^
Place of living	Not in Attica	15	71.4	6	28.6	0.008^c^
	Attica	110	93.2	8	6.8	
education	High school	23	74.2	8	25.8	0.005^c^
	university	85	94.4	5	5.6	
	Master	20	95.2	1	4.8	
occupation	Household- unemployed	20	90.9	2	9.1	1.000^c^
	Public Servant	27	90.0	3	10.0	
	Private Employee	63	90.0	7	10.0	
	Merchants. tradesmen	16	88.9	2	11.1	
	1	76	92.7	6	7.3	0.351^c^
No of children	2	39	84.8	7	15.2	
	≥3	12	92.3	1	7.7	
labour	caesarian	76	91.6	7	8.4	0.499^a^
	normal	52	88.1	7	11.9	
Family support	less	28	84.8	5	15.2	0.317^c^
	Too much	99	91.7	9	8.3	

^a^Pearson’s χ2 test;^b^Student’s t-test; ^c^Fisher’s exact test

Table 3 shows SF-36 mean scores. Mean value of Physical Component Summary was 36.1 (±9.2) and Mental Component Summary was 53.8 (±9.5).

**Table 3:** SF-36 mean scores

	min	max	mean	SD	p for normality test
Physical Functioning	0.0	100.0	58.8	28.2	0.318
Role-Physical (RP)	0.0	100.0	33.3	38.3	0.001
Bodily Pain	0.0	100.0	44.9	25.7	0.092
General Health (GH)	40.0	100.0	81.5	12.7	0.079
Vitality	0.0	95.0	59.6	18.5	0.561
Social Functioning	0.0	100.0	65.7	26.1	0.101
Role-Emotional (RE)	0.0	100.0	73.3	37.5	0.088
Mental Health (MH)	28.0	100.0	79.4	13.8	0.411
Physical Component Summary	18.1	58.2	36.1	9.2	0.638
Mental Component Summary	26.4	70.4	53.8	9.5	0.520

*p* value for Kolmogorov-Smirnov test

Multivariate linear regression analysis with stepwise method was applied with dependent variable the scores of «Physical Functioning», «Role –physical», «Bodily pain» and «General health» (Table 4). Therefore, Relationship with their husband and type of labor were found to be independent variables that correlated significantly with the dimension «Physical Functioning». Participants with very good relationship with their husband had higher score in dimension «Physical Functioning» by 13.13 points compared with participants who had poor. Those who had normal birth had higher score in dimension «Physical Functioning» at 14.58 points, compared with participants who had a caesarean. Depression was found to be independently associated with the dimension «Role-Physical». Women with postpartum depression scored 24.27 lower in «Role-Physical». Depression was found to be independently associated with the dimension «Bodily pain». Women with postpartum depression scored 15.60 lower in «Bodily pain» dimension. Depression was found to be associated significantly with the dimension «General Health».

**Table 4:** Multivariate linear regression analysis results (stepwise method) for Physical Functioning, Role-Physical, Bodily pain and General Health

		β	SE	P
*Physical Functioning*				
Relationship with their husband	poor	0.00^a^		
	Very good	13.13	6.48	0.045
Labor	Caesarian	0.00		
	Normal	14.58	4.92	0.004
*Role-Physical*				
Depression symptoms	no	0.00		
	yes	−24.27	11.07	0.030
*Bodily pain*				
Depression symptoms	no	0.00		
	yes	−15.60	7.20	0.032
*General Health*				
Depression symptoms	no	0.00		
	yes	−0.12	0.03	0.002

^a^indicates reference category; analysis was based on log-transformed values

Multivariate linear regression analysis results with dependent variable the scores of Vitality, Social functioning, Role Emotional and Mental Health are shown in Table 5. Regression analysis revealed that Women with postpartum depression scored 14.18 lower in dimension of «Vitality». In addition, Place of living, education and depression correlate significantly with «social functioning. In particular, participants living in Attica scored 12.42 higher in «Social Functioning». Participants with university education scored 17.35 higher in «Social Functioning» compared to those with master education, whereas participants with higher school education scored 22.35 higher in «Social Functioning» compared to participants with master education. Also, participants with postpartum depression scored 25.68 lower in social functioning and 38.25 lower in Role Emotional. Furthermore, presence of depression and not very good relationships with their husband were independently associated with lower score on Emotional Wellbeing. (Table 5).

**Table 5:** Multivariate linear regression analysis results (stepwise method) for Vitality, Social functioning, Role Emotional and Mental Health

		β	SE	P
*Vitality*				
Depression	no	0.00^a^		
	yes	−14.18	5.12	0.006
*Social functioning*				
Place of living	Outside Attica	0.00		
	Attica	12.42	5.95	0.039
Educational level	master	0.00		
	Higher school	22.35	7.33	0.003
	university	17.35	6.21	0.006
Depression symptoms	no	0.00		
	yes	−25.68	7.21	0.001
Role – Emotional				
Depression symptoms	no	0.00		
	yes	−38.25	10.79	0.001
*Emotional Wellbeing*				
Depression symptoms	no	0.00		
	yes	−16.82	3.42	<0.001
Relationship with their husband	poor	0.00		
	Very good	9.98	2.73	<0.001

^a^indicates reference category

Table 6 shows the linear regression analyses results for Physical Component Summary and Mental Component Summary. Those who had normal labor scored 4.51 higher in Physical Component Summary than those who had a caesarian. Furthermore, women with postpartum depression scored 12.58 lower in Mental Component Summary than those who hadn’t. (Table 6).

**Table 6:** Multivariate linear regression analysis results (stepwise method) for Physical and Mental Component Summary

		β	SE	P
*Physical Component Summary*				
Labor	Caesarian	0.00^a^		
	Normal	4.51	1.76	0.012
*Mental Component Summary*				
Depression	no	0.00		
	yes	−12.58	2.63	<0.001

^a^indicates reference category

## Discussion

The current study assessed the quality of life of a sample of mothers in Greece, in order to investigate the association between postpartum depression and quality of life (QoL). Quality of life is a concept affected by culture and social systems [12, 17], thus it was found important to assess it in the Greek context in order to explore any differences presented in that context.

Findings of the current study revealed that 9.9% of participants, experienced post-partum depression symptoms. The number of the studies that have been conducted in the specific field in Greece is limited. In the study of Gonidakis et al. [27] in a sample of 402 Greek women, 19.8% experienced PPD during the first 6 months after delivery. However, in the specific study the prevalence was different when women were approached after one week, one, three and six months [27]. When new mothers were approached one week after delivery, that as a time point in close to our study, only 6.8% were found to have PPD symptoms [27]. In another study among 205 Greek women, 14.5% reported PPD symptoms 2–3 days after delivery [28]. However, in the specific study a cut-off score of EPDS ≥14, which is consistent to the screening of major depression symptoms, was used [28]. In another study conducted among 1039 women in Greece, prevalence of PPD was 13.6% (EPDS ≥13) at 8 weeks postpartum [29]. By taking into account the above, we could say that our findings are not easily comparable with the results of other studies conducted in Greece, since a number of differences exist in the cut-off criteria of EPDS and assessment periods. Additionally, comparison with other countries’ rates is limited, since PPD prevalence is highly variable worldwide [6].

As far as the correlations found between postpartum depression and demographics of the participants, significant correlations were found between the place of residence and symptoms of postpartum depression, since women outside of Attica indicated higher levels of postpartum depression symptoms (*p* = 0.008) than women living in Attica. Previous studies have showed that place of residence may be related with postpartum depression [30]. According to the results of a study conducted among 29,405 women who lived in metropolitan Sydney in Australia, new mothers who were residents of the suburbs expressed higher postpartum depression symptoms, due to poor social-environmental conditions [30]. In our study, living outside Attica does not indicate poor living conditions such as poverty or criminality. However, since Attica is the metropolitan area of Athens, which is the capital of Greece, new mothers that took part in our study and were residents of Athens, have delivered in a place near their home, had better access to any service that they may require in the near future and a more expanded social network, including friends and relatives, that could provide them social support after childbirth.

In addition, in the current study, the level of education was found to be significantly correlated with postpartum depression symptoms, since women without tertiary education, experienced higher levels of postpartum depression symptoms (*p* = 0.005). However, our sample was not representative, since most of the participants had undertaken tertiary education which may affect the specific result.

As far as the quality of life assessment with the SF-36 scale, women who delivered naturally had worse subjective physical functioning, compared to those who had caesarean (*P* = 0.001) which is not in line with the findings of previous studies [31]. Also, women who had a very good relationship with their husband had better Physical Functioning compared to those who had poor (*P* = 0.017), which is in line with previous findings [27, 32]. Women who had symptoms of postpartum depression had significantly lower scores in the dimension “General Health” (P = 0.01), indicating worse physical role, compared to those who had no symptoms of postpartum depression.

Similar, but not the same, were the findings of a prospective study of Sadat et al. [4], in Iran, which showed that depression symptoms may affect most of quality of life fields among women, postpartum. More specifically, in our study depression symptoms were found to be associated significantly with five out of eight sub scales of SF-36 (General Health, Role-Physical, Bodily pain, Vitality and Emotional Wellbeing), while in Sadat et al. study [4] there were significant negative correlations between PPD scores and seven out of SF-36 subscales (except Role-Physical). However, in Sadat et al. study assessments of both PPD and quality of life were made prospectively in the second and fourth month postpartum, while in our study the assessment was made between third and fourth day after delivery.

Taking into consideration the aforementioned findings, there is a need for further longitudinal studies in order to explore the quality of life of those women on a long-term basis. Research data in the specific field may orient clinicians attention and provision of care to particular quality of life domains that seems to be more affected from PPD presence.

However, during the interpretation of the data, we must take into account the fact that during the past 7 years the financial recession and the political instabilities in Greece have created a difficult situation from which the majority of people have been affected. The county lost more than the 25% of its gross domestic product (GDP), [33], while a reduction in health spending of more than a third in real terms was made since the beginning of the crisis in 2009 [34]. Unemployment rates reached 26.15% in 2014 and remain high (compared to 7.8% in 2008) and proportion of population at risk of social exclusion because of poverty rose from 28.1% in 2008 to 36% in 2014 [35]. According to the findings of a systematic review concerning the impact of the financial crisis in Greek population, in which the results of 39 studies conducted in this period were included and considered for further analyses, crisis had a lot of consequences including increased rates in mental health problems, especially depression, and suicides, while a deterioration in of self-rated health was also reported [36].

As far as the strengths of the current research, those lie to the size of the study population and the uniformity of their ethnicity. Concerning the sample size, as we already mentioned in methods section, the research team approached all women who had given birth at the clinic and met the inclusion criteria during a period of three months, with a high response rate. Therefore, although we did not follow a random selection method, the fact that all women approached may be taken into account as a minimum probability sampling criterion. As far as the ethnicity, given that reports for PPD’s prevalence display a great variability due to cross-cultural variables [6], data from different countries and ethnicities may contribute to the better research and the comparison of the diversities of the prevalence. Undoubtedly, there were also some limitations. First and foremost is the design chosen, since correlational type of design, on the one hand provides the opportunity for correlations but correlations do not refer to individuals. Further, correlations do not identify causes. Finally, the sample was not representative of the broader population, since 77% of the participants had tertiary level qualification.

## Conclusions

Postpartum depression symptoms are associated with quality of life of women after pregnancy and therefore constitutes a powerful predictor of quality of life. Holistic care models targeting the improvement of both physical and social functioning, that according to the findings are both affected from PPD symptoms and individualized care depended on the way of delivery (normal or caesarean) and the presence or absence of social recourses (e.g. presence of a good relationship with husband) may prove to be helpful for maintaining a good level of quality of life in women with PPD symptoms.

## Abbreviations

ANOVA: Analysis of Variance
BP: bodily pain
EPDS: Edinburgh Postnatal Depression Scale
EW: emotional wellbeing
GH: general health
PF: Physical Functioning
PPD: Postpartum depression
QoL: Quality of Life
RE: role-emotional
RP: role-limitation due to physical problems or role physical
SF: Social Functioning
SF-36: Greek version of Medical Outcomes study-short form SF-36
SPSS: Statistical Package for the Social Sciences
VT: vitality
